# Selection of reference genes for the quantitative real-time PCR normalization of gene expression in *Isatis indigotica* fortune

**DOI:** 10.1186/s12867-019-0126-y

**Published:** 2019-03-25

**Authors:** Renjun Qu, Yujing Miao, Yingjing Cui, Yiwen Cao, Ying Zhou, Xiaoqing Tang, Jie Yang, Fangquan Wang

**Affiliations:** 1 College of Horticulture, Nanjing Agricultural University, Nanjing, 210095 China; 2 Institute of Food Crops, Jiangsu Academy Agriculture Sciences, Nanjing, 210014 China

**Keywords:** *Isatis indigotica*, qRT-PCR, Reference gene, Gene expression, Normalization

## Abstract

**Background:**

*Isatis indigotica*, a traditional Chinese medicine, produces a variety of active ingredients. However, little is known about the key genes and corresponding expression profiling involved in the biosynthesis pathways of these ingredients. Quantitative real-time polymerase chain reaction (qRT-PCR) is a powerful, commonly-used method for gene expression analysis, but the accuracy of the quantitative data produced depends on the appropriate selection of reference genes.

**Results:**

In this study, the systematic analysis of the reference genes was performed for quantitative real-Time PCR normalization in *I. indigotica*. We selected nine candidate reference genes, including six traditional housekeeping genes (*ACT*, *α*-*TUB*, *β*-*TUB*, *UBC*, *CYP*, and *EF1*-*α*), and three newly stable internal control genes (*MUB*, *TIP41*, and *RPL*) from a transcriptome dataset of *I. indigotica*, and evaluated their expression stabilities in different tissues (root, stem, leaf, and petiole) and leaves exposed to three abiotic treatments (low-nitrogen, ABA, and MeJA) using geNorm, NormFinder, BestKeeper, and comprehensive RefFind algorithms. The results demonstrated that *MUB* and *EF1*-*α* were the two most stable reference genes for all samples. *TIP41* as the optimal reference gene for low-nitrogen stress and MeJA treatment, while *ACT* had the highest ranking for ABA treatment and *CYP* was the most suitable for different tissues.

**Conclusions:**

The results revealed that the selection and validation of appropriate reference genes for normalizing data is mandatory to acquire accurate quantification results. The necessity of specific internal control for specific conditions was also emphasized. Furthermore, this work will provide valuable information to enhance further research in gene function and molecular biology on *I. indigotica* and other related species.

**Electronic supplementary material:**

The online version of this article (10.1186/s12867-019-0126-y) contains supplementary material, which is available to authorized users.

## Background

*Isatis indigotica* fortune, a biennial herbaceous plant belonging to the Cruciferae family [1], is widely distributed and cultivated across China. Its dried roots (*Radix Isatidis*), namely “Ban-Lan-Gen”, have been used as a traditional Chinese medicine to treat fever, influenza, epidemic hepatitis, and bacterial infection for thousands of years [2]. During the 2003 SARS (severe acute respiratory syndrome) outbreak in China, Ban-Lan-Gen played a significant role in preventing SARS through its antiviral effect [3]. The dried leaves of *I. indigotica*, named “Da-Qing-Ye”, can be used to produce “Qing-Dai” (*Indigo naturalis*), a dark blue powder that is used to treat psoriasis [4], colonic inflammation [5], leukemia [6], and cancer [7]. To date, numerous phytochemical studies of this herb have led to the isolation of virous bioactive constituents, including alkaloids, epigoitrin, phenolic acids, flavonoids, and lignans, of which indole alkaloids are the dominant compounds. These ingredients have been reported in numerous studies due to their antiviral [8], antibacterial [9], anti-inflammatory [10] and anticancer [11] properties. However, most of them have a low abundance in plants, for example, indican, isatin, indirubin, and indigotin account for 1.16–43.6 μg/g DW (dry weight), 0.30–3.45 μg/g DW, 1.01–34.4 μg/g DW, and 1.45–18.7 μg/g DW, respectively [12]. These active compounds are secondary metabolites that accumulate during normal plant growth or exposure to environmental stresses [13]. It is necessary to elucidate the biosynthetic pathway of *I. indigotica* under various stresses to increase the content of active ingredients. With the development of high-throughput sequencing technology, many candidate genes involved in the biosynthesis of active ingredients have been obtained from the transcriptome database [14, 15]. Therefore, an understanding of functional gene expression profiling will provide us with better insight into the metabolic pathway and the regulatory mechanism operating under stresses in this medical herb.

With its high sensitivity, accuracy, and specificity as well as its high-throughput characteristic, the quantitative real-time polymerase chain reaction (qRT-PCR) has become the most powerful and reliable molecular technique for gene expression analysis in a wide range of biological research areas [16, 17]. However, the quantitative results are often affected by several error sources, such as the amount of starting material, the RNA integrity, reverse transcription, and qRT-PCR amplification [18]. To obtain accurate qRT-PCR analysis results, it is crucial to normalize the raw gene expression data. The use of stable reference genes as normalization factors to minimize these errors has become the most common approach [18]. Housekeeping genes, or genes involved in basic metabolism, such as actin (*ACT*), glyceraldehyde-3-phosphate dehydrogenase (*GAPDH*), tubulin (*TUB*), and elongation factor 1 alpha (*EF*-*1α*) have traditionally served as references in plant science, because they were believed to be consistently expressed across various tissues, developmental stages, and treatments [19, 20]. Nevertheless, numerous studies have reported that the transcription level of commonly-used housekeeping genes shows unacceptable variability under different experimental conditions [21]. If inappropriate reference genes are selected for normalization, the noise of the expressing assay will be increased, and thus, misinterpretation of the results will appear [22]. Consequently, it is essential to systematically evaluate potential reference genes to ensure that they are appropriate for a specific experimental condition [23]. To date, IGG (http://icg.big.ac.cn), a wiki-driven knowledgebase that collects internal reference genes for diverse species, has been integrating a comprehensive collection of more than 150 plants [24], such as *Arabidopsis* [25], cucumber [26], wheat [27], rice [28], *Artemisia annua* [29], and *Panax ginseng* [30]. However, it has not been used for the systematic selection of a reference gene for qRT-PCR analysis in *I. indigotica* under hormone treatment or low-nitrogen stress, a factor that impedes functional gene studies.

High-throughput mRNA sequencing (RNA-Seq), a transcriptome profiling-based deep-sequencing technology approach, has paved the way for the use of transcriptome analysis in various species at an amazing scale and speed [31]. With recent advances, RNA-Seq can reveal novel genes, carry out tissue-specific alternative splicing, and identify differentially expressed genes [32]. Meanwhile, plant transcriptome data have commonly been used to search for appropriate reference genes through this technique [33]. Excel-based tools, such as geNorm [34], NormFinder [35] and BestKeeper [36], have been developed to select the most suitable reference genes from a set of biological samples under investigation to be used in an expression stability analysis. Besides this, the optimal gene should be evaluated with genes of interest to obtain reliable results. As a non-model species, few previous studies have been conducted on *I. indigotica* at a molecular level. Lignans, a component of *I. indigotica* that are produced by the phenylpropanoid pathway, are important chemical ingredients that exhibit various biological activities. Cinnamoyl coenzyme A reductase (CCR) is one of the key enzymes involved in the biosynthesis of lignin monomers, and the expression of the *IiCCR* gene shows prominent diversity in response to hormonal stress [37]. Therefore, *IiCCR* may be used to demonstrate that the reference genes are reliable under various experimental conditions.

In this study, nine common candidate reference genes were selected based on the transcriptome libraries of *I. indigotica* (SRR1051997) to determine appropriate reference genes for qRT-PCR normalization in different plant tissues, and under low-nitrogen stress and exposure to hormonal stimuli (ABA and MeJA). Moreover, the expression level of one target gene, *IiCCR*, was assayed to verify the reliability of the proposed reference genes. Finally, the results will provide the basis for further research in exploring gene expression profiling under different experimental conditions.

## Results

### Selecting reference genes based on transcriptome data and the performance of amplification primers

Based on previous reports in *Arabidopsis* [25], cucumber [26] and wheat [27], we selected nine genes as candidate genes by mining the *Isatis indigotica* transcriptome data [14]. The details of gene symbol, gene ID, gene name, Arabidopsis ortholog no, and characteristics of PCR amplification in *Isatis indigotica* are shown in Table 1. Subsequently, qRT-PCR primers were designed, and their specificity was determined using gel electrophoresis and a melting curve. The 2% agarose gel electrophoresis showed that only one amplicon corresponding to the expected fragment size was obtained after PCR amplification in all candidate reference genes (Additional file 1: Figure S1), and a single amplification peak was present on the melting curve for all primer sets (Additional file 1: Figure S2). qRT-PCR amplification efficiencies of 91% and 109% with correlation coefficients (*R*^2^) ranging from 0.9859 to 0.9987 were calculated based on a standard curve assay generated from amplification with a series of cDNA dilutions (Table 1).

**Table 1:** Candidate reference genes, primer sequences, and characteristics of PCR amplification in *Isatis indigotica*

Gene symbol	Gene ID	Gene name	Primer: forward/reverse	Arabidopsis ortholog no.	Identify (%)	Amplicon size (bp)	E (%)	*R* ^2^
*ACT*	GARR01013235.1	Actin	GCTCACGGAAGCACCT CGACCACTAGCGTAAAGT	At3g53750	95	128	91	0.9984
*UBC*	GARR01010405.1	Ubiquitin-conjugating enzyme	TTTGCTGGAAAGGGAC TTGGAGGTTTGAAAGGAT	At1g50490	92	105	107	0.9944
*α*-*TUB*	GARR01008456.1	Alpha-tubulin	GAGCCTTTGTTCATTG CAACCTCCTCATAATCC	At1g50010	83	102	109	0.9968
*β*- *TUB*	GARR01020827.1	Beta-tubulin	TAAAGAAGTGGACGAAC CCCTTAGGAGGAATGT	At5g12250	88	111	96	0.9987
*EF1*-*α*	GARR01019351.1	Elongation factor 1-α	GCCGATTGTGCTGTCC GTGGCATCCATCTTGTTA	At1g07930	96	149	94	0.9859
*MUB*	GARR01001157.1	Membrane-anchored ubiquitin-fold protein	TTTCCCGATGCTACAA ACCTGAGGCTGAATGA	At5g15460	90	215	109	0.9969
*CYP*	GARR01006610.1	Cyclophilin	GGCAAGACAGTTCCTA TGATTCTCCACCCATA	At2g29960	89	177	99	0.9975
*RPL*	GARR01002832.1	Ribosomal protein L18	CAAGGGCTAGGATTGA TAAGGTTTGGAGTGGC	At5g27850	90	172	106	0.9976
*TIP41*	GARR01011419.1	TIP41-like family protein	AACACTTGAGCAGCAA GTATGGCGTCGTAAAA	At4g34270	93	173	105	0.9980

### Reference gene expression levels

**Fig. 1:**
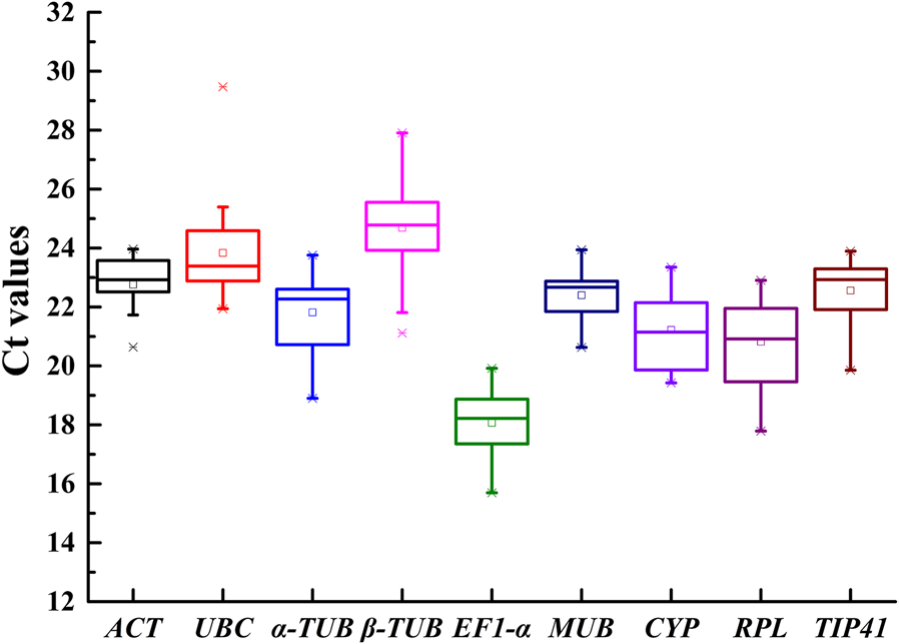
Cycle threshold value (Ct) of nine candidate reference genes across all samples. The box chart indicates the interquartile range. The outer box represents the 25th to 75th percentiles, and the inner box represents the mean values. The lower and upper dashes depict the minimum and maximum values. The line across the box is the median. The asterisks represent the outliers

The transcript abundances of the nine reference genes were determined from their mean cycle threshold value values (Ct) and varied from 15 to 29, with lower Ct values corresponding to higher expression abundance. Among all candidates, *EF1*-*α* had the highest transcript level with the lowest mean Ct value of 18.07 ± 1.23 (mean ± SD), followed by *RPL* (20.82 ± 1.58), *CYP* (21.23 ± 1.30), *α*-*TUB* (21.82 ± 1.35), *MUB* (22.40 ± 0.89), and *TIP41* (22.56 ± 1.04). *β*-*TUB* displayed the lowest expression level with a mean Ct value of 24.69 ± 1.89, followed by *UBC* (23.84 ± 1.73), and *ACT* (22.76 ± 1.05) (Fig. 1, Additional file 1: Table S1). Genes with Ct values with large SDs had more variable expression compared to these with lower SDs. *MUB* showed the smallest variation in gene expression (22.40 ± 0.89), while *β*-*TUB* showed the most variable level of expression.

### Analysis of expression stability of the candidate reference genes

The expression stability of nine candidates across different sample sets was evaluated and ranked using four different computational algorithms including geNorm, NormFinder, BestKeeper, and RefFinder.

### geNorm analysis

**Fig. 2:**
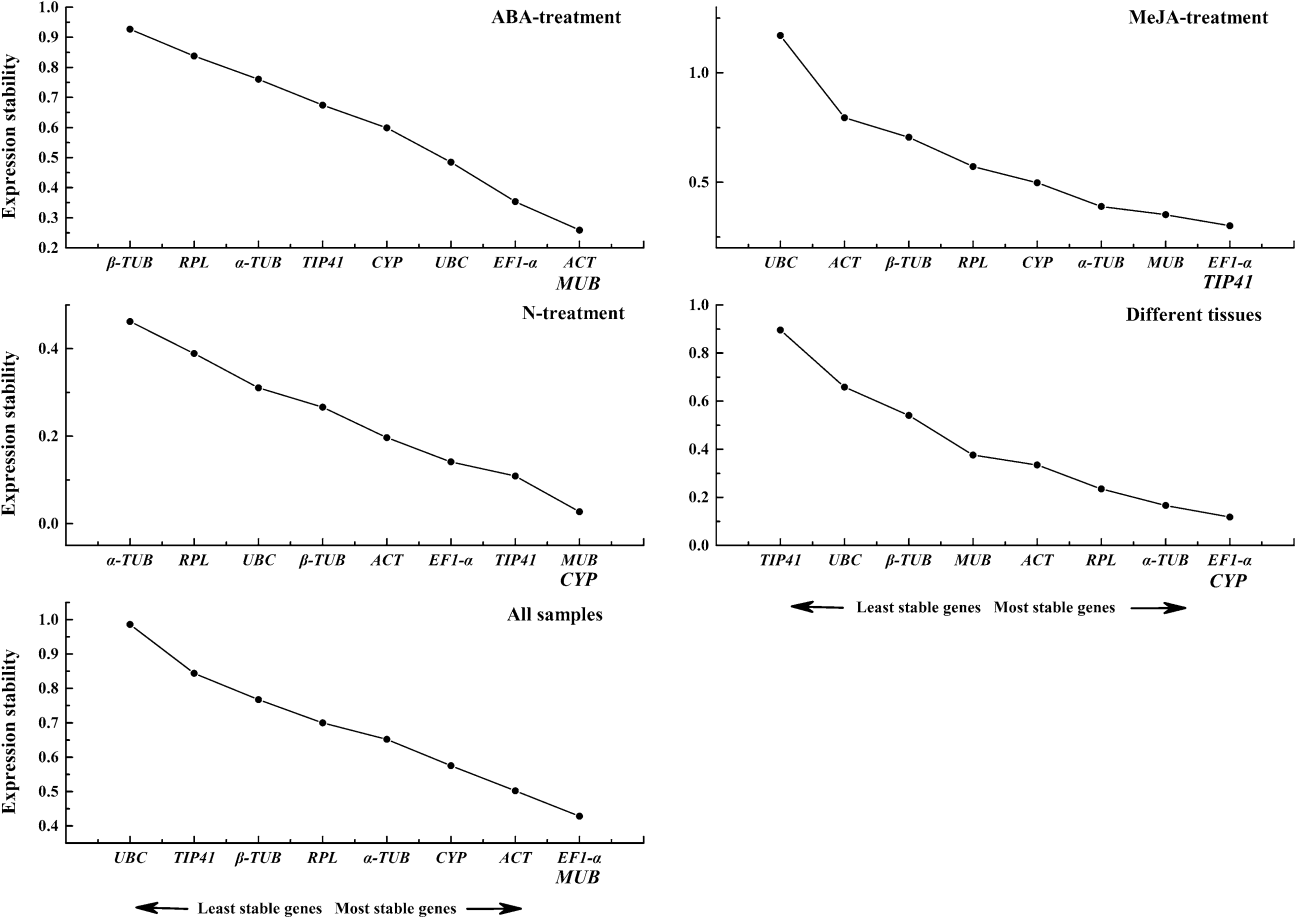
Expression stability values (M) of the nine candidate reference gene evaluated by geNorm. A lower M value indicates more stable expression. The most stable genes are on the right and the least stable genes are on the left

The expression stability values (*M*) calculated by geNorm were used to evaluate the stability of the nine proposed reference genes by comparing the average variation of each gene to all others. An *M* value of 1.5 was used as a threshold for expression stability, so the gene with the lowest *M* value was recognized as the most stable reference gene and vice versa [34]. Based on the above criteria, when all the samples of different tissues and abiotic stresses were combined, *EF1*-*α* and *MUB* had the lowest *M* value, whereas *UBC* had the highest value, indicating that *EF1*-*α* and *MUB* possessed the most stable expression, and *UBC* was the most variably expressed. For the ABA stress set, the two most stable genes for normalization were *ACT* and *MUB* with the minimum *M* value, and *β*-*TUB* was the least stable gene. In the MeJA stress set, *EF1*-*α* and *TIP41* ranked as the two most stable gene and *UBC* was the least stable one. In the set of samples under low-nitrogen stress, *MUB* and *CYP* were the top two stable gene and *α*-*TUB* was the most unstable candidate. Finally, in the various tissues set, *EF1*-*α* and *CYP* were the most stable gene, and *TIP41* was the least stable gene (Fig. 2).

**Fig. 3:**
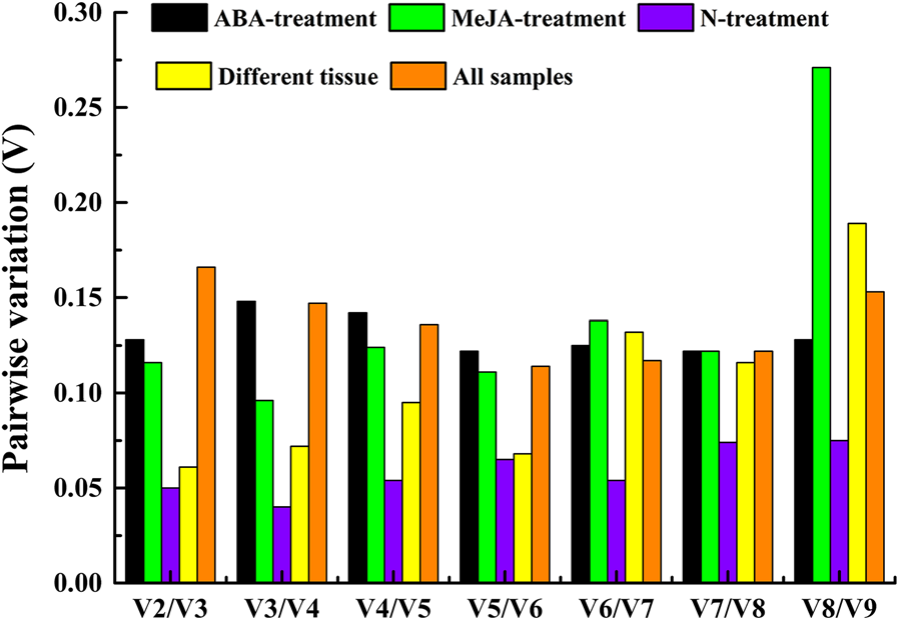
Pairwise variation (V) of the nine candidate reference genes calculated by geNorm to determine the optimal number of reference genes for accurate normalization. The threshold used was 0.15

To obtain the optimal number of genes needed for qRT-PCR normalization, the average pairwise variation (*Vn*/*Vn *+ 1) between two sequential normalization factors (NF_n_ and NF_n+1_) was calculated using the geNorm programme. The cutoff value of *Vn*/*Vn *+ 1 < 0.15 indicated that *n* stable reference genes are enough to obtain accurate results [34]. Under exposure to ABA stress, MeJA stress, low-nitrogen stress, and different tissues, the *V2*/*V3* value was already below 0.15, indicating that two reference genes were sufficient for accurate normalization. When all the samples were taken together, the pairwise variation (*V2/V3*) was 0.166, while *V3/V4* was 0.147, indicating that the addition of a third reference gene had a significant effect on the results (Fig. 3).

### NormFinder analysis

The optimal normalization gene among these candidates was determined by NormFinder according to their stability values, as shown in Table 2. A lower stability value indicated more stable expression of a gene. In the subset of all samples, *MUB*, *EF1*-*α*, and *ACT* were identified as the three most stable reference genes, while *UBC* showed higher variation. *TIP41* and *EF1*-*α* were identified as the two most stable reference genes under low-nitrogen stress, while *TIP41* was the least stable gene in different tissues. Under MeJA stress, *MUB* and *α*-*TUB* were suggested to be the most stable genes, and *UBC* was the least stable one. Consistent with the geNorm analysis, *ACT* was shown to be the most stable gene under ABA stress, and *EF1*-*α* and *CYP* were the two most stable genes in different tissues.

**Table 2:** Expression stability of the nine candidate reference genes as calculated by NormFinder

Rank	ABA treatment		MeJA treatment		N treatment		Different tissues		All samples	
	Gene	Stability	Gene	Stability	Gene	Stability	Gene	Stability	Gene	Stability
1	*ACT*	0.116	*MUB*	0.144	*TIP41*	0.049	*EF1*-*α*	0.041	*MUB*	0.188
2	*EF1*-*α*	0.184	*α*-*TUB*	0.153	*EF1*-*α*	0.075	*CYP*	0.041	*EF1*-*α*	0.191
3	*MUB*	0.247	*EF1*-*α*	0.271	*CYP*	0.098	*α*-*TUB*	0.084	*ACT*	0.327
4	*UBC*	0.275	*TIP41*	0.382	*MUB*	0.123	*ACT*	0.127	*CYP*	0.366
5	*CYP*	0.510	*β*-*TUB*	0.409	*ACT*	0.128	*MUB*	0.134	*α*-*TUB*	0.433
6	*RPL*	0.551	*RPL*	0.511	*β*-*TUB*	0.279	*RPL*	0.232	*RPL*	0.440
7	*α*-*TUB*	0.592	*CYP*	0.574	*UBC*	0.322	*β*-*TUB*	0.662	*β*-*TUB*	0.541
8	*TIP41*	0.633	*ACT*	0.614	*RPL*	0.364	*UBC*	0.753	*TIP41*	0.694
9	*β*-*TUB*	0.782	*UBC*	1.683	*α*-*TUB*	0.464	*TIP41*	1.172	*UBC*	0.942

### BestKeeper analysis

The BestKeeper algorithm was used to rank the stability of the reference genes according to the standard deviations (SD) and coefficients of variance (CV) of their Ct values, which are listed in Table 3. Higher stability was indicated by a lower CV ± SD value. Ct values with SDs of less than 1 were considered to have an acceptable range of variation. *MUB* (1.31 ± 0.29) and *TIP41* (1.45 ± 0.34) were the most stable genes for expression normalization in the ABA stress set; *TIP41* (1.05 ± 0.25) and *MUB* (1.49 ± 0.35) in MeJA stress; *CYP* (0.29 ± 0.05) and *TIP41* (0.29 ± 0.06) in low-nitrogen stress; and *ACT* (1.62 ± 0.34) and *MUB* (1.66 ± 0.35) in different tissues. As for the all-samples set, *MUB* showed the highest expression stability, which is consistent with the geNorm and NormFinder results.

**Table 3:** Expression stability of the nine candidate reference genes, as calculated by BestKeeper

Rank	ABA treatment		MeJA treatment		N treatment		Different tissues		All samples	
	Gene	CV ± SD	Gene	CV ± SD	Gene	CV ± SD	Gene	CV ± SD	Gene	CV ± SD
1	*MUB*	1.31 ± 0.29	*TIP41*	1.05 ± 0.25	*CYP*	0.29 ± 0.05	*ACT*	1.62 ± 0.34	*MUB*	3.21 ± 0.72
2	*TIP41*	1.45 ± 0.34	*MUB*	1.49 ± 0.35	*TIP41*	0.29 ± 0.06	*MUB*	1.66 ± 0.35	*ACT*	3.52 ± 0.80
3	*ACT*	1.72 ± 0.40	*EF1*-*α*	1.56 ± 0.30	*MUB*	0.32 ± 0.07	*CYP*	2.96 ± 0.60	*TIP41*	3.78 ± 0.85
4	*CYP*	2.42 ± 0.53	*α*-*TUB*	1.99 ± 0.44	*EF1*-*α*	0.47 ± 0.08	*α*-*TUB*	3.15 ± 0.63	*α*-*TUB*	4.99 ± 1.08
5	*EF1*-*α*	2.50 ± 0.47	*ACT*	2.30 ± 0.53	*ACT*	0.94 ± 0.21	*TIP41*	3.24 ± 0.69	*UBC*	4.96 ± 1.18
6	*UBC*	3.22 ± 0.78	*CYP*	2.45 ± 0.55	*β*-*TUB*	0.97 ± 0.24	*EF1*-*α*	3.67 ± 0.60	*CYP*	5.29 ± 1.12
7	*α*-*TUB*	4.20 ± .94	*RPL*	2.85 ± 0.62	*UBC*	1.18 ± 0.27	*RPL*	4.19 ± 0.78	*EF1*-*α*	5.69 ± 1.03
8	*RPL*	4.30 ± 0.89	*β*-*TUB*	2.88 ± 0.75	*RPL*	1.85 ± 0.41	*β*-*TUB*	4.32 ± 0.95	*β*-*TUB*	5.50 ± 1.35
9	*β*-*TUB*	4.40 ± 1.11	*UBC*	7.33 ± 1.83	*α*-*TUB*	2.08 ± 0.47	*UBC*	5.27 ± 1.22	*RPL*	6.38 ± 1.32

### RefFinder analysis

As shown in Table 4, the RefFinder program, which integrates geNorm, NormFinder and BestKeeper, was used to generate a comprehensive ranking of the nine candidate reference genes. In this process, *MUB* and *EF1*-*α* were ranked as the top two genes of the total sample. *TIP41* was suggested to be the most stable gene under MeJA stress and low-nitrogen stress, while it was unstably expressed under different tissues. *CYP* comprehensively ranked first in different tissues. Under ABA stress, *ACT* was the most stable. The transcript abundances of *UBC* were extremely unstable in all samples, different tissues, and under MeJA stress. *α*-*TUB* was found to be unstable under low-nitrogen stress, while for ABA stress, *β*-*TUB* was the least stable gene.

**Table 4:** Expression stability of the nine candidate reference genes based on the RefFinder analysis

Experimental treatments									
Total		ABA treatment		MeJA treatment		N treatment		Different tissues	
Most	Least	Most	Least	Most	Least	Most	Least	Most	Least
*MUB*	*UBC*	*ACT*	*β*-*TUB*	*TIP41*	*UBC*	*TIP41*	*α*-*TUB*	*CYP*	*UBC*
*EF1*-*α*		*MUB*		*EF1*-*α*		*CYP*		*EF1*-*α*	
*ACT*									

### Validation of the selected reference genes

**Fig. 4:**
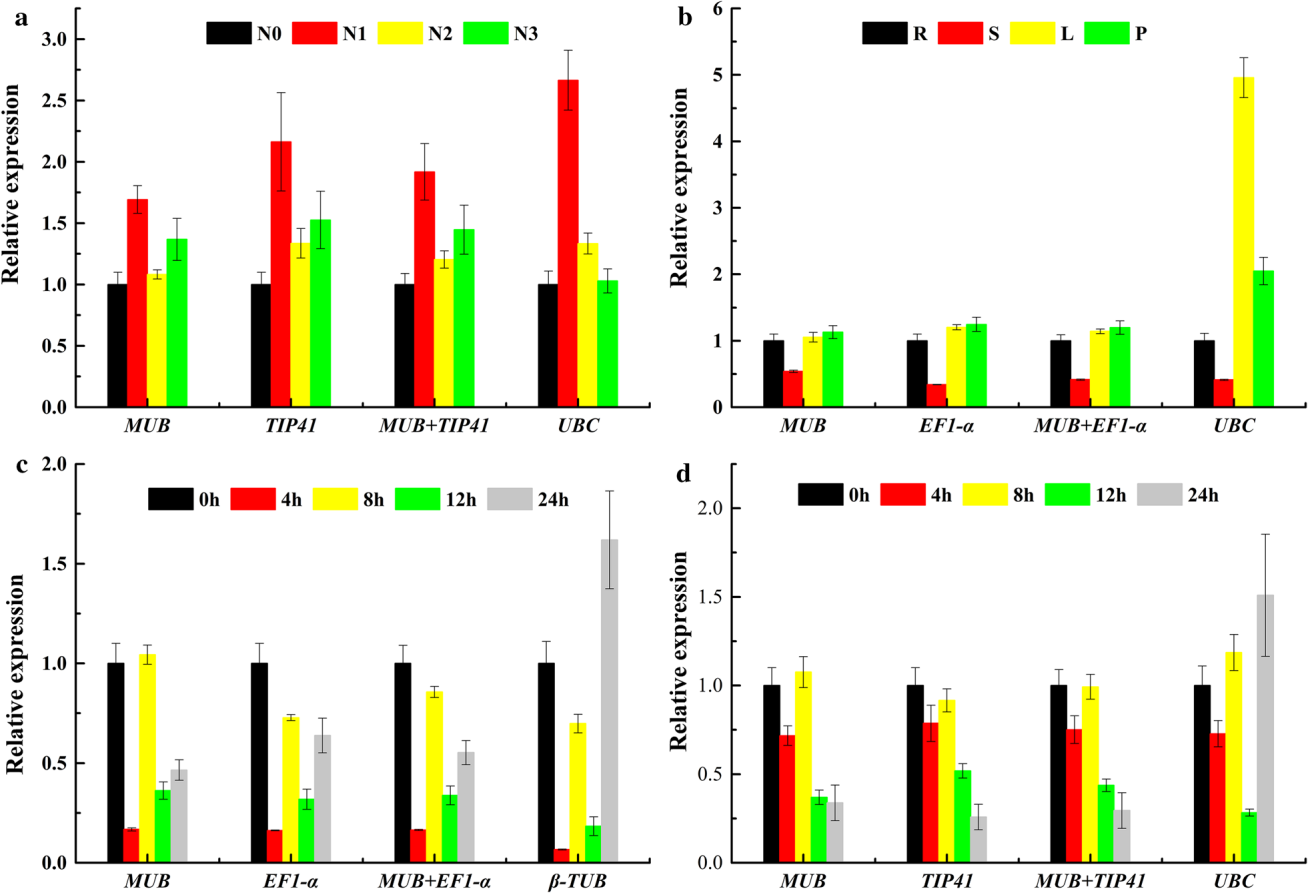
Relative expression of IiCCR using the selected reference genes. The results were normalized using the selected stable reference genes (singly or in combination) and the unstable genes in sample sets across treatment with **a** N, **b** different tissues, **c** ABA, and **d** MeJA. The bars indicate the standard error (± SE) evaluated from three biological replicates

To validate the utility of the proposed reference genes in these four experiments, the relative expression level of *IiCCR* was detected using the most stable (*MUB* or *TIP41* were used alone or *MUB* was combined with *TIP41* for low-nitrogen stress and MeJA stress, *MUB*, *EF1*-*α*, or their combination for ABA stress and different tissues) and least stable (*UBC* for low-nitrogen stress, MeJA stress and different tissues, *β*-*TUB* for ABA stress) reference genes as calibrators. In low-nitrogen stress, the highest expression level of *IiCCR* was detected in N1, followed by N3 and N2, and then in N0. By contrast, there was a distinct discrepancy when using *UBC* as the least stable reference gene (Fig. 4a). We noticed that *IiCCR* has a similar expression pattern normalized by the optimal reference gene and the most unstable reference visibly differed (Fig. 4b). The *IiCCR* expression level was significantly upregulated at 0 h and 8 h under ABA treatment when *MUB*, and *EF1*-*α* were used as the internal control genes. However, it was at 0 h and 24 h when *β*-*TUB* was used (Fig. 4c). The normalization results of the *IiCCR* expression level in MeJA treatment were consistent when using the two optimal genes (*MUB* and *TIP41*) as calibrators, while significant deviations appeared when normalized by the worst reference gene, *UBC* (Fig. 4d). These outcomes proved that accurately normalize gene expression, it is important to validate reference genes with stale expression under diverse experimental conditions.

## Discussion

The growth and development of plants is challenged by unsuitable environmental factors, such as salinity, drought, UV stress, and pathogen infection due to habitat restriction [13]. Being sessile, it is necessary for plants to evolve a series of defense and/or adaption mechanisms. Among these, plant secondary metabolites are known to play major roles in the adaptation of plants to their environments and in conferring protection against stress conditions [38]. Furthermore, the metabolites are unique sources for active pharmaceuticals, cosmetics, and food additives [39]. Unfortunately, the yield of plant secondary metabolites is so low that they cannot meet the increasing demand of the market. To increase their production, the biosynthesis pathways and key gene expression profiling related to the biosynthesis of the secondary metabolites need to first be elucidated. Then, we will be able to establish a better understanding of gene functions [40]. qRT-PCR has emerged as a broadly accepted method for gene expression analysis due to its accuracy, high-throughput, and sensitivity [18]. Nevertheless, selecting reference genes from the literature without systematic validation could cause inaccurate qRT-PCR results [22]. Hence, the selection and validation of appropriate reference genes for normalizing data is mandatory to acquire accurate quantification results under various experimental conditions for a given species.

High-throughput sequencing technologies with fast development have provided a highly effective method to study plant transcriptomics [41], plant epigenomics [42], and plant genomics [43]. Moreover, the creation of large data sets and gene expression data by sequencing are regarded as an abundant source for reference gene selection, especially for non-model plants. Therefore, *I. indigotica* large-scale transcriptome data (SRR1051997) can serve as a gene pool to identify potential internal control genes. Systematic and comprehensive evaluation of nine reference genes was performed in different tissues of *I. indigotica* and in leaves subjected to various treatments. The results showed that a single amplification peak presented on the melting curve images, so all the primer sets had quite good specificity (Additional file 1: Figure S2). Additionally, the qRT-PCR performance of tested reference genes suggested high amplification efficiency values (close to 100%) (Table 1). The statements mentioned above justify that these primers worked as expected and were reliable for further analyses of the stability of candidate reference genes.

Three statistical algorithms (geNorm, NormFinder, and BestKeeper) were used to analyze the stability of these candidates. However, the stability rankings obtained from the algorithms were not identical. The results of geNorm and NormFinder were similar for some conditions, but discrepancies occurred for the orders ranked by BestKeeper. For instance, in the ABA treatment, geNorm and NormFinder calculated *ACT* to be the most stable gene, while it was ranked moderately by BestKeeper. This apparent variation was probably due to the different calculation principles in the three statistical algorithms [44]. To obtain consistent results, a comprehensive online tool, RefFinder, which integrates the three algorithms, has been widely applied to generate a final comprehensive ranking of reference gene expression [45]. In the current study, the RefFinder analysis, identified *MUB* as the most stable gene in the all-samples set. Meanwhile, *UBC*, *α*-*TUB* and *β*-*TUB* had relatively poor expression stability values, which is similar to previous results in *Artemisia annua* L [29] and *Brassica napus* [46].

Traditionally, classical housekeeping genes have been regarded as stably expressed at various development stages and under different treatments. However, an increasing number of studies is showing that the expression stabilities of most of these genes actually have great variation [22, 45]. Therefore, the use of housekeeping genes as references must be validated under specific conditions. In this study, six traditional housekeeping genes, which are involved in the cytoskeleton (*ACT*, *α*-*TUB* and *β*-*TUB*), post-translational modification (*UBC* and *CYP*), and ribosomal structure and biogenesis (*EF1*-*α*), were shown to display dramatic differences in expression patterns under conditions of low-nitrogen stress, hormonal stimuli, and in different tissues. *CYP* was the most stable reference gene in different tissues of *I. indigotica* but exhibited relatively low stability in *Lycoris aurea* [19]. *EF1*-*α* ranked neither as the top, nor as the least suitable gene under four experimental conditions. *α*-*TUB* and *β*-*TUB* of the tubulin gene family are used as reference genes in many species, such as *Capsicum annuum* L. [47], *Cynodon dactylon* under cold stress [48], and *Eremosparton songoricum* under various stress conditions [49], but in our study, they, along with *UBC*, always displayed the least stable expression pattern. In addition, *ACT* was ranked first in ABA treatment and was also the most stable gene in the all-samples set. Consistent with the result in *Lilium davidii var. unicolor*, *ACT* also showed strong stability in *I. indigotica* [50]; however, *ACT* is not appropriate for gene normalization in different organs of *Salix matsudana* [51].

Compared with the traditional housekeeping genes, the newly reported reference genes performed better in gene normalization under specific conditions [19, 44]. In this research, three newly reported reference genes, *MUB*, *TIP41*, and *RPL*, were analyzed. MUBs are membrane-anchored ubiquitin-fold proteins, which are thought to play a crucial role in diverse signaling cascades [52]. The corresponding genes are universally expressed in the tissues of many plants, animals, and fungi [53]. In *Salix matsudana*, *MUB* was shown to be highly stable under salt and drought stress conditions [51]. In the current study, we also found *MUB* to be the most suitable gene in the all-samples subset. The *RPL* gene also served as a stable reference gene [54], but it showed less stable expression patterns under almost all tested conditions in *I. indigotica*. Similar to *Lycoris aurea* [19], *TIP41* was revealed to be the optimal reference gene for MeJA stress. As for *I. indigotica*, *TIP41* also ranked first under low-nitrogen stress and was selected as the best reference gene. Previous studies have indicated that *TIP41* has fairly stable expression during salt stress in oilseed rape as well as at different developmental stages and in various tissues of *Arabidopsis* plants [46, 55]. In addition, *TIP41* was not only validated as a reliable internal control gene under abiotic stress in chickpeas [56], it was shown to be suitable for cucumber plants under various degrees of nitrogen nutrition [57].

The expression patterns of a target gene *IiCCR* were examined using the two most stable and least stable reference genes to further confirm the stabilities of reference genes. The results showed that the *IiCCR* displayed a consistent expression pattern in response to low-nitrogen stress, hormonal stimuli and in different tissues when *MUB* was used as an internal control, either singly or in combination with *TIP41* or *EF1*-*α*. However, severe variance appeared when the least stable genes, *UBC* or *β*-*TUB*, were used for normalization. Our results were consistent with previous studies, which reported that the use of unstable reference genes for qRT-PCR analysis resulted in significant variation in target gene amplification profiles, resulting in the misinterpretation of expression data [58]. Consequently, it is extremely important to systematically select reference genes to accurately measure the target genes’ expression levels.

## Conclusions

The selection of suitable reference genes is a prerequisite to quantifying gene expression by qRT-PCR. In this study, a series of candidate reference genes were systematically validated to normalize gene expression during *I. indigotica*’s response to various conditions. Three prevalently-used algorithms (geNorm, NormFinder, and Bestkeeper) were adopted to analyze the expression stability of the nine candidates. RefFind produced the final comprehensive ranking, showing that the optimal reference genes were *MUB* and *EF1*-*α* across all samples; *TIP41* and *CYP* under low-nitrogen stress; *EF1*-*α* and *TIP41* under MeJA stress; *CYP* and *EF1*-*α* in different tissues; and *ACT* and *MUB* under ABA stress. The reference genes identified as the least stable, *β*-*TUB* and *UBC*, are not recommended for the normalization of transcripts. The qRT-PCR of *IiCCR* was used to validate the reliability of these results, and the selected reference genes were shown to significantly reduce the error rate in gene quantification. The results obtained from the present work will help to further increase the accuracy of normalization in qRT-PCR analysis and will facilitate gene expression studies in *I. indigotica*.

## Methods

### Plant materials and treatments

Seeds of *Isatis indigotica*, collected from ShanXi province in Northern China, were used in this study. The seeds were soaked in tap water to wash away the empty seeds floating on the water. The plump seeds were sown in plastic pots filled with a mixture of perlite and vermiculite (ratio, 1:1; v/v) and maintained in the greenhouse of Nanjing Agricultural University (118°51′ E; 32°1′ N), Nanjing, China. After germination, seedlings were irrigated with 1/4 strength Hoagland’s solution once a week before being subjected to different experimental treatments 6 weeks later.

For the hormonal stimuli, the leaves of the seedling were sprayed with 100 μM abscisic acid (ABA treatment) or 100 μM methyl jasmonate (MeJA treatment) and then collected at 0, 4, 8, 12, and 24 h. Low-nitrogen level stress was produced by irrigating the seedlings with a solution comprising five concentration levels of nitrogen for 1 week, which all included 1 mM KH_2_PO_4_, 2 mM MgSO_4_, 2.5 mM CaCl_2_, 46 μM H_3_BO_3_, 9 μM MnCl·4H_2_O, 0.32 μM CuSO_4_·5H_2_O, 0.76 μM ZnSO_4_·7H_2_O, 0.5 μM H_2_MoO_4_, and 20 μM FeSO_4_ (EDTA Na_2_) [59] in addition to 0 mM KNO_3_, 2.5 mM KNO_3_, 5 mM KNO_3_, and 10 mM KNO_3_. Root, stem, leaf, and petiole tissues were collected from untreated seedlings. Three biological repeats were collected for all samples from each treatment, immediately frozen in liquid nitrogen, and stored at − 80 °C for total RNA extraction.

### Total RNA isolation and cDNA synthesis

Total RNA from each sample was extracted using the RNAprep Pure Plant Kit (TIANGEN) and treated with DNase I to avoid genomic DNA contamination, in accordance with the kit instructions. The RNA concentration and purity were quantified using a Colibri spectrophotometer (Berthold Detection). The integrity of the purified RNA samples was examined by 1.5% (p/v) agarose gel electrophoresis. Samples were used for cDNA synthesis at absorption ratios of A260/A280 = 1.9–2.1 and A260/A230 ≥ 2.0. A first strand cDNA synthesis reaction was carried out and transcribed from 2 μg total RNA and 1 μg oligo-dT in a final volume of 20 μL using the PrimeScript™ 1st strand cDNA Synthesis Kit (TaKaRa) by following the manufacturer’s protocols. The final cDNA samples were diluted five-fold with RNAase-free water and then stored at − 20 °C until further analysis.

### Selection of candidate reference genes and primer design

The candidate genes selected in the present study served as reference genes that were previously reported as suitable for gene expression normalization in other model plants and similar species subject to different experimental conditions. Their names were used to search the *I. indigotica* transcriptome library (SRR1051997) and the genes that were extensively expressed in organizations were selected (Table 1). Moreover, to ensure the reliability and correctness of the proposed reference genes, we BLAST-searched the nucleotide sequences of candidate genes against the Arabidopsis genome database to identify their homologs in *I. indigotica*. Based on the unigene sequences (Additional file 1: File S1), specific primers were designed using primer 3 software (http://bioinfo.ut.ee/primer3-0.4.0/) with the following criteria: melting temperature (TM) 58–62 °C, GC content 40–65%, primer length 16–20 bp, and amplicon length 100–220 bp (Table 1). Self-complementarity and hair-pin structures were avoided. The primer specificity was judged by the agarose gel electrophoresis of the PCR amplification products (Additional file 1: Figure S1) and observed via melting curves (Additional file 1: Figure S2).

### PCR and qRT-PCR analysis

PCR amplification was performed in a total volume of 20 μL, containing 3.6 μL of ddH_2_O, 2 μL of five-fold diluted cDNA, 10 μL of 2× PCR buffer, 2 μL of dNTPs (2 mM), 1 μL of each primer (10 mM), and 0.4 μL of KOD Fx (1.0 U/μL). The PCR program was as follows: 5 min at 94 °C, 35 cycles of 10 s at 98 °C, 30 s at 60 °C, and 30 s at 68 °C, followed by 5 min extension at 68 °C. The PCR products were run on 2% agarose gel electrophoresis (Additional file 1: Figure S1). The qRT-PCR was conducted in 96-well plates with an ABI 7500 real-time PCR system (Applied Biosystems) using the SYBR^®^ Green I (Biouniquer). The reaction mixture contained 2 μL of five-fold diluted cDNA, 2 μL of each primer (10 mM), 0.4 μL of 50 × ROX1 and 10 μL of RealTime PCR Master Mix to give a final volume of 20 μL. The program for qRT-PCR was set as 10 min at 95 °C, 40 cycles of 15 s at 95 °C, and 30 s at 60 °C. The melting curves were recorded in each reaction by constantly raising the temperature from 65 to 90 °C (Additional file 1: Figure S2). Each sample was run with three technical replicates, and every plate included one no template control (NTC) to monitor possible DNA contamination. The threshold cycle (Ct) was measured automatically. A standard curve was generated with five-fold series dilution of the mixed cDNA of all samples to calculate the PCR efficiency (E) and correlation coefficient (*R*^2^). The PCR amplification efficiency (E) of each primer pair was calculated by the curve slope using E = [5^(−1/slope)^ − 1] × 100% [51].

### Data analysis to evaluate the expression stability of the reference genes

The stability and suitability of the nine selected reference genes were evaluated by three algorithms, geNorm [34], Normfinder [35], and Bestkeeper [36], across all experimental sets. Finally, RefFinder (http://150.216.56.64/referencegene.php) integrated the three algorithms to obtain an overall ranking. For geNorm and Normfinder, the mean Ct value of three biological repeats from each gene was converted into the relative expression level using the formula 2^−ΔCt^ (ΔCt = Ct value of each sample − the lowest Ct value) [60]. For BestKeeper, the mean Ct value was imported into the program directly. Stability measures (M) of the candidate genes were calculated with the geNorm algorithm. Stepwise exclusion of the gene with the highest M (least stable gene) value was used to rank the analyzed genes. Subsequently, the pairwise variation (*Vn/Vn *+ 1) values calculated by the geNorm were used to determine the optimal number of candidate reference genes; a value below 0.15 indicated that no additional reference gene was required. NormFinder evaluated the genes’ expression stability by assessing intra and intergroup variation in a given sample set, offering a ranking in which the highest stability (S) value represented the least stable gene [61]. BestKeeper was used to calculate the standard deviation (SD) and coefficient of variation (CV) of the average Ct values. Analyzed genes with a standard deviation (SD) > 1 were considered to be unacceptable reference genes, and the gene with the lowest CV ± SD value was the most stable one.

To obtain a more accurate expression analysis, the 54 samples were divided into four experimental sets and analyzed individually: 15 samples from the ABA-induced *I. indigotica* leaves (set 1, ABA treatment); 15 samples from the MeJA-induced *I. indigotica* leaves (set 2, MeJA treatment); 12 samples from the low-nitrogen stressed *I. indigotica* leaves (set 3, N treatment); and 12 samples from different tissues (roots, stems, leaves, and petioles) of *I. indigotica* (set 4, different tissues). In addition, the stability of the four sets together and that of each variety was analyzed.

### Validation of reference gene stability

To identify the stability of the reference genes selected in this study, the expression level of *IiCCR*, a gene involved in the lignin monomers biosynthesis pathway [37], was detected with qRT-PCR analysis. The expression patterns of *IiCCR* in samples of *I. indigotica* under low-nitrogen stress, MeJA treatment, ABA treatment, and in different tissues were normalized using two most and one least stable reference genes, respectively, as recommended by RefFinder. The 2^−ΔΔCT^ method, a commonly used method to analyze the relative exchange in gene expression, was used to calculate the relative expression data of the target gene [62]. Three technical replicates were performed for each biological sample.

## Additional file

**Additional file 1: Table S1.** Ct values of the 9 candidate reference genes. **Figure S1.** Specificity of primer pairs for qRT-PCR amplification. **Figure S2.** Melting curves of the 9 candidate reference genes showing single peaks. **File S1.** Sequences of nine candidate reference genes.

## Abbreviations

*ACT*: actin
*UBC*: ubiquitin-conjugating enzyme
*α-TUB*: alpha-tubulin
*β-TUB*: beta-tubulin
*EF1-α*: elongation factor 1-α
*MUB*: membrane-anchored ubiquitin-fold protein
*CYP*: cyclophilin
*RPL*: ribosomal protein L18
*TIP41*: TIP41-like family protein
