# Supplementary material for: Selection of reference genes for the quantitative real-time PCR normalization of gene expression in Isatis indigotica fortune
Source: BMC Mol Biol. 2019 Mar 25;20:9. doi: 10.1186/s12867-019-0126-y (PMC6434783; doi:10.1186/s12867-019-0126-y)
Supplement: Supplementary file 1 — Additional file 1: Table S1. Ct values of the 9 candidate reference genes. Figure S1. Specificity of primer pairs for qRT-PCR amplification. Figure S2. Melting curves of the 9 candidate reference genes showing single peaks. File S1. Sequences of nine candidate reference genes. [file 12867_2019_126_MOESM1_ESM.docx]

**Table S1.** Ct values of the 9 candidate reference genes.

|  |  | *ACT* | *UBC* | *α-TUB* | *β-TUB* | *EF1-α* | *MUB* | *CYP* | *RPL* | *TIP41* |
| --- | --- | --- | --- | --- | --- | --- | --- | --- | --- | --- |
| ABA-treatment | 0h | 23.58 ± 0.01 | 24.79 ± 0.01 | 23.76 ± 0.04 | 25.34 ± 0.05 | 18.69 ± 0.03 | 22.03 ± 0.04 | 22.70 ± 0.05 | 20.71 ± 0.02 | 23.84 ± 0.04 |
|  | 4h | 22.78 ± 0.13 | 22.89 ± 0.04 | 21.83 ± 0.02 | 23.88 ± 0.06 | 18.51 ± 0.04 | 21.91 ± 0.04 | 20.85 ± 0.03 | 19.90 ± 0.03 | 23.72 ± 0.03 |
|  | 8h | 22.90 ± 0.04 | 23.37 ± 0.03 | 20.59 ± 0.03 | 24.52 ± 0.01 | 17.93 ± 0.01 | 21.79 ± 0.03 | 21.94 ± 0.02 | 19.42 ± 0.02 | 22.93 ± 0.04 |
|  | 12h | 23.58 ± 0.08 | 24.59 ± 0.03 | 22.83 ± 0.09 | 24.55 ± 0.04 | 18.70 ± 0.09 | 22.23 ± 0.19 | 21.33 ± 0.05 | 20.54 ± 0.07 | 23.38 ± 0.01 |
|  | 24h | 23.88 ± 0.03 | 24.87 ± 0.05 | 22.91 ± 0.02 | 27.91 ± 0.03 | 19.92 ± 0.03 | 22.81 ± 0.12 | 21.93 ± 0.07 | 22.90 ± 0.01 | 22.95 ± 0.03 |
| MeJA-treatment | 0h | 23.22 ± 0.01 | 24.49 ± 0.02 | 22.52 ± 0.06 | 25.40 ± 0.06 | 19.49 ± 0.02 | 23.94 ± 0.01 | 22.76 ± 0.02 | 21.93 ± 0.04 | 23.89 ± 0.01 |
|  | 4h | 22.64 ± 0.01 | 23.78 ± 0.02 | 22.42 ± 0.08 | 26.64 ± 0.05 | 19.67 ± 0.07 | 23.21 ± 0.02 | 23.35 ± 0.82 | 22.91 ± 0.04 | 23.29 ± 0.03 |
|  | 8h | 22.51 ± 0.03 | 23.89 ± 0.03 | 21.95 ± 0.01 | 25.85 ± 0.07 | 18.84 ± 0.01 | 23.20 ± 0.03 | 23.21 ± 0.03 | 21.91 ± 0.04 | 22.92 ± 0.02 |
|  | 12h | 23.96 ± 0.02 | 22.89 ± 0.05 | 21.02 ± 0.12 | 25.55 ± 0.06 | 18.87 ± 0.07 | 22.72 ± 0.02 | 21.76 ± 0.04 | 20.24 ± 0.06 | 23.16 ± 0.04 |
|  | 24h | 23.84 ± 0.13 | 29.47 ± 0.02 | 22.25 ± 0.08 | 27.70 ± 0.01 | 19.16 ± 0.12 | 23.60 ± 0.05 | 22.15 ± 0.02 | 21.96 ± 0.07 | 23.16 ± 0.03 |
| N-treatment | N0 | 22.89 ± 0.06 | 22.83 ± 0.10 | 22.29 ± 0.28 | 24.75 ± 0.16 | 17.44 ± 0.09 | 22.87 ± 0.05 | 19.94 ± 0.04 | 21.13 ± 0.03 | 21.89 ± 0.02 |
|  | N1 | 23.32 ± 0.04 | 23.33 ± 0.06 | 22.54 ± 0.19 | 24.81 ± 0.08 | 17.36 ± 0.01 | 22.72 ± 0.03 | 19.82 ± 0.03 | 21.93 ± 0.03 | 22.09 ± 0.12 |
|  | N2 | 22.95 ± 0.01 | 22.88 ± 0.02 | 22.60 ± 0.03 | 25.13 ± 0.02 | 17.60 ± 0.29 | 22.63 ± 0.03 | 19.76 ± 0.01 | 22.42 ± 0.03 | 21.94 ± 0.04 |
|  | N3 | 23.39 ± 0.06 | 22.31 ± 0.06 | 23.74 ± 0.19 | 24.26 ± 0.04 | 17.35 ± 0.05 | 22.76 ± 0.08 | 19.86 ± 0.05 | 22.26 ± 0.04 | 21.94 ± 0.05 |
| Different tissues | R | 20.90 ± 0.03 | 21.94 ± 0.02 | 19.61 ± 0.02 | 21.81 ± 0.02 | 15.94 ± 0.02 | 20.63 ± 0.03 | 19.73 ± 0.01 | 17.97 ± 0.05 | 21.28 ± 0.02 |
|  | S | 20.64 ± 0.08 | 21.97 ± 0.01 | 18.90 ± 0.05 | 21.12 ± 0.05 | 15.69 ± 0.03 | 21.05 ± 0.04 | 19.42 ± 0.01 | 17.79 ± 0.05 | 21.88 ± 0.01 |
|  | L | 21.73 ± 0.04 | 25.40 ± 0.43 | 20.72 ± 0.03 | 23.93 ± 0.02 | 17.35 ± 0.09 | 21.85 ± 0.08 | 20.96 ± 0.02 | 19.46 ± 0.03 | 19.85 ± 0.03 |
|  | P | 20.91 ± 0.06 | 23.40 ± 0.05 | 20.29 ± 0.11 | 21.23 ± 0.05 | 16.69 ± 0.09 | 21.23 ± 0.07 | 20.58 ± 0.29 | 19.42 ± 0.17 | 21.91 ± 0.04 |
|  | mean+sd | 22.76 ± 1.05 | 23.84±1.73 | 21.82±1.35 | 24.69±1.89 | 18.07±1.23 | 22.40±0.89 | 21.23±1.30 | 20.82±1.58 | 22.56±1.04 |


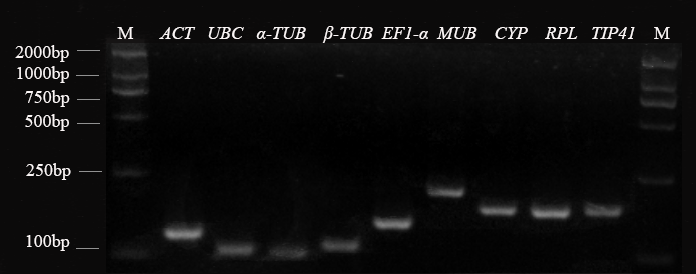


**Figure S1.** Specificity of primer pairs for qRT-PCR amplification. The 2% agarose gel electrophoresis showing a single product of the expected size for each candidate reference gene. M represents the DNA size marker.

**
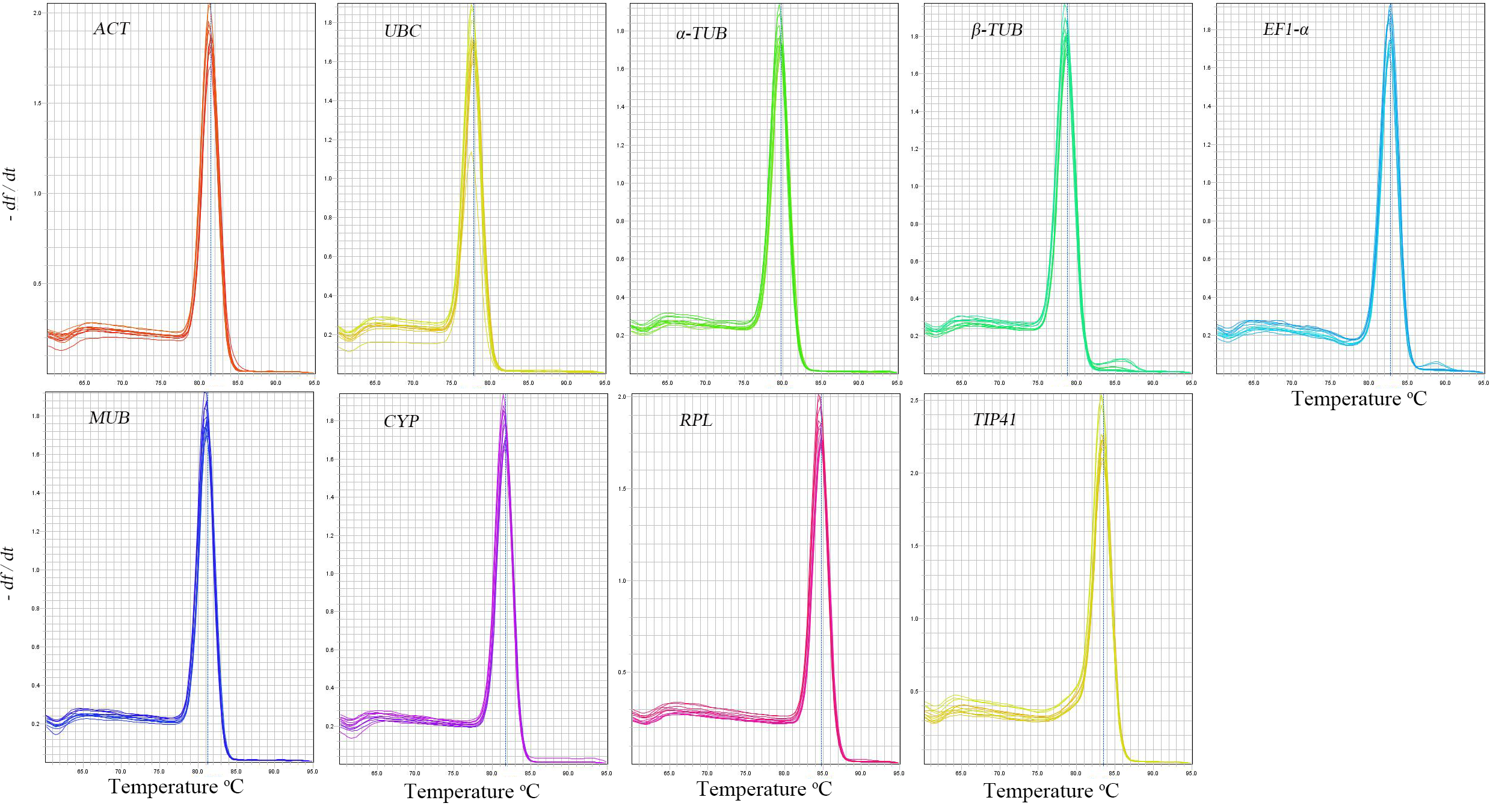
**

**Figure S2.** Melting curves of the 9candidate reference genes showing single peaks.

**File S1.** Sequences of nine candidate reference genes

**1. *ACT***

ATGGCTGACGGTGAAGACATTCAACCTCTTGTTTGCGACAATGGAACTGGAATGGTTAAGGCTGGTTTTGCTGGAGATGATGCACCGAGGGCTGTGTTTCCAAGTATCGTAGGCCGTCCTCGTCACACCGGAGTGATGGTGGGGATGGGACAGAAGGATGCATACGTTGGTGATGAAGCTCAATCCAAGCGTGGTATTCTAACTCTCAAATACCCAATCGAGCACGGTATTGTCAACAATTGGGATGACATGGAGAAAATCTGGCATCATACTTTCTACAATGAGCTCCGTGTTGCTCCCGAGGAGCATCCGATCCTGCTCACGGAAGCACCTCTCAACCCAAAGGCTAACCGTGAGAAGATGACTCAAATCATGTTTGAAACTTTCAATGCTCCGGCCATGTATGTAGCCATTCAAGCTGTCCTGTCACTTTACGCTAGTGGTCGTACTACAGGTATTGTACTCGACTCTGGGGATGGTGTGAGTCACACAGTCCCTATCTATGAGGGTTACGCTCTTCCGCATGCCATCCTACGTCTCGATCTTGCAGGTCGTGACCTCACAGATGCTCTGATGAAGATCCTCACTGAGCGTGGTTACTCTTTCACCACTACAGCTGAGCGTGAAATTGTCAGAGACATAAAGGAGAAGCTTTGCTACATTGCTCTGGACTATGAGCAGGAGCTTGAGACGGCTAAAACCAGCTCTGCTGTGGAGAAGAACTATGAGTTACCTGATGGGCAAGTGATCACCATTGGATCTGAGCGGTTCCGTTGCCCAGAGGTTCTTTATCAGCCATCTATGATCGGTATGGAGAATGCCGGAATCCATGAAACCACCTACAACTCCATAATGAAATGTGATGTCGACATCAGAAAGGACTTGTATGGTAACATTGTGCTCAGTGGTGGAACCACTATGTTCCCTGGAATTGCTGACAGAATGAGCAAAGAGATCACCGCTTTGGCACCGAGCAGCATGAAGATCAAAGTCGTTGCACCGCCTGAGAGGAAATACTCCGTCTGGATTGGAGGATCCATCTTGGCCTCTCTCAGTACCTTCCAGCAGATGTGGATCGCAAAGGCGGAATATGACGAGTCAGGCCCGTCGATTGTTCACCGGAAATGCTTT

**2.** ***UBC***

ATGGCTGCCGTTAATGGGTACCAGGGGAATACTCCGGCGGATCCTCCGGCTTCAAATGGATCAAAGCAATCTGCGCCGCCGACTAAGACTGTTGACAGCCAATCTGTTCTCAAAAGGCTGCAATCTGAACTTATGGGTTTGATGATGGGTGGTGACCCGGGAATCTCTGCTTTCCCAGAGGAAGACAACATATTTTGCTGGAAAGGGACAATAACAGGAAGCAAAGAGACTGTCTTTGAAGGAACTGAGTACAGACTCTCACTCTCTTTCTCCAATGACTATCCTTTCAAACCTCCAAAGATCAAGTTCGAGACTTGTTGCTTCCACCCCAATGTTGATGTCTATGGCAATATCTGCTTGGACATTCTTCAGGATAAATGGTCATCTGCTTACGATGTGAGGACAATACTACTATCGATTCAGAGCCTTCTGGGAGAACCGAACATCAGCTCACCATTGAACACTCAAGCAGCTCAGCTCTGGGGCAACCAAGAAGAGTATAGGAAGATGGTTGAGAAGCTCTACAAGCCTCCCAGTGCA

**3.** ***α-TUB***

ATGCTCTCCTCCTACGCTCCTGTCATCTCAGCAGAGAAAGCGTACCATGAGCAGCTTTCAGTTGCAGAGATCACAAACACTGCGTTCGAGCCTTCCTCTATGATGGCTAAGTGTGACCCGCGCCACGGAAAGTACATGGCTTGTTGTCTAATGTACAGAGGAGATGTTGTCCCGAAGGATGTCAATGCTGCGGTTGGAACCATCAAGACAAAACGAACCATCCAGTTCGTGGATTGGTGTCCGACCGGGTTTAAGTGCGGTATCAATTACCAGCCTCCTACTGTTGTCCCGGGTGGTGATCTTGCTAAAGTCCAGAGAGCTGTGTGTATGATCTCGAACTCGACGAGTGTCGCTGAAGTCTTCTCGAGGATTGACCATAAGTTTGATCTGATGTACGCGAAGAGAGCCTTTGTTCATTGGTATGTTGGTGAAGGTATGGAGGAAGGTGAGTTCTCTGAGGCTCGTGAGGATCTTGCTGCACTTGAGAAGGATTATGAGGAGGTTGGTGCAGAAGGTGATGATGGAGAGGAAGACGAAGGAGAAGAGTAT

**4. *β-TUB***

ATGTTCCGTGGCAAAATGAGCACTAAAGAAGTGGACGAACAGATGATCAACGTCCAGAACAAGAACTCTTCTTACTTTGTTGAATGGATCCCCAACAATGTCAAGTCCAGTGTCTGTGACATTCCTCCTAAGGGTCTCTCAATGGCATCGACCTTTGTTGGTAACTCGACTTCGATCCAGGAGATGTTTAGGCGGGTGAGCGAGCAGTTCACGGCTATGTTCAGGAGAAAAGCTTTCTTGCATTGGTACACA

**5. *EF1-α***

ATGGGTAAAGAGAAGTTTCACATCAACATTGTGGTCATTGGCCACGTCGATTCTGGAAAATCGACAACCACTGGTCACTTGATCTACAAGCTTGGTGGTATTGACAAGCGTGTGATCGAGAGGTTCGAGAAGGAAGCTGCTGAGATGAACAAGAGGTCTTTCAAGTACGCGTGGGTCTTGGACAAACTTAAGGCCGAGCGTGAGCGTGGTATCACCATTGACATTGCTCTCTGGAAGTTCGAGACCACCAAGTACTACTGCACAGTCATTGATGCTCCTGGACATCGTGATTTCATCAAGAACATGATTACTGGTACCTCCCAGGCCGATTGTGCTGTCCTCATCATTGACTCCACCACTGGTGGTTTTGAAGCTGGTATCTCCAAGGATGGTCAGACCCGTGAGCACGCTCTTCTTGCTTTCACCCTCGGTGTCAAGCAAATGATTTGCTGTTGTAACAAGATGGATGCCACTACCCCCAAATACTCCAAGGCTAGGTACGATGAGATCATCAAGGAGGTGTCTTCCTACTTGAAGAAGGTTGGGTACAACCCTGACAAAATCCCATTCGTCCCCATCTCTGGGTTCGAGGGTGACAACATGATTGAGAGGTCCACCAACCTTGACTGGTACAAGGGACCAACTCTCCTCGAGGCTCTTGACCAGATCAACGAGCCCAAGAGGCCCTCAGACAAGCCCCTCCGTCTCCCACTTCAGGATGTGTACAAGATCGGTGGT

**6. *MUB***

ATGGCAGAGGTAAAGGATCATTTAGAGATCAAGTTCCGGTTGACTGATGGTTCAGATATCGGTCCTAAATCGTTTCCCGATGCTACAACGGTTTCAACATTGAAAGAAACTGTGGTTGCTCAATGGCCAAGAGAAAAGGAGAATGGGCCAAAGACAGTGAAAGATGTGAAATTGATAAGCGCGGGTAGAATATTGGAGAACAACAAGACAGTTGGAGATTGCAGGAGTGCTGTCTGTAATCTCTCAGGTGCTGTCACCACAATGCATGTTATCATTCAGCCTCAGGTTATTGAAAAAGAAAAGAAGAAGAAGCCTAAAGGTGATCTGAAACAGAACAAATGCGTCTGTTTATGTTTTGGAAATCGGTTT

**7. *CYP***

ATGGCGAGACTAAGCTCTATTCTATTTGCGACGCTACTCCTTTTCGCTGCTCTAGCTTTAATCCAGGCAAAGGAAGATCCGAAGGAAATCACTCACAAGGTTTACTTCGATGTAGAGATCGACGGGAAAGAAGCTGGTAGAGTTGTGATTGGCCTATTTGGCAAGACAGTTCCTAAAACTGCAGAAAACTTCAGAGCTCTTTGCACAGGGGAGAAAGGTGTGGGGAAGAGCGGGAAACCTCTACACTACAAGGGAAGCAAGTTCCATAGAATCATTCCCAGCTTTATGATCCAAGGTGGTGACTTCACGCACGGAAATGGTATGGGTGGAGAATCAATCTACGGTCAGAAGTTTGCAGATGAGAACTTCAAGCTGAAGCACACCGGACCAGGTTTTCTTTCGATGGCAAACTCTGGAGAAGACACAAACGGTTCGCAGTTTTTCATCACGACCGTGACAACAAGCTGGTTAGATGGAAGGCATGTTGTGTTTGGTAAAGTGGTTCAAGGAATGGATGTTGTGTACAAGATTGAAGCTGAGGGTAAGCAAAGCGGGACTCCTAAGAGCAAAGTCGTTATTGCAGACAGTGGAGAGCTTCCTCTT

**8. *RPL***

ATGGGTATCGATCTTATCGCCGGAGGTAAGAGCAAGAAGACCAAAAGGACAGCTCCAAAGTCCGATGATGTCTACCTCAAGCTTCTCGTCAAGCTATACCGGTTTTTGGTAAGGAGAACTGGCAGCAAGTTCGATGCTGTGATCCTTAAGAGGCTTTTCATGAGCAAAGTCAACAAAGCTCCTCTTTCACTCTCCAGACTCGTTGAATTCATGAAGGGCAAGGATGGTAAGATTGCTGTGTTGGTTGGGACAATAACTGATGATTTGAGAGTGCACGAGATCCCTGCCATGAAGGTTACTGCCTTGAGGTTTACAGAGAGAGCAAGGGCTAGGATTGAGAAAGCTGGTGGAGAGTGCTTAACCTTTGACCAGCTTGCCCTCATTGCTCCATTGGGCCAGAACACGGTTCTCCTTAGAGGACCCAAGAACTCTCGTGAGGCAGTGAAGCACTTTGGTCCTGCGCCTGGTGTGCCACACAGCCACTCCAAACCTTATGTTAGGTCTAAGGGAAGGAAGTTTGAGAAAGCTAGAGGAAAGCGAAAGAGTCGTGGATTCAAGGTC

**9. *TIP41***

ATGGAGACGGAGGTCGATAAGGAGATTCTAAAATCTGCCGGGGCGGAGCTTCTTCCCGACGGACGTCGAGGTTTGCGCATCCATGACTGGGAGATCGAAACCCTCCGCGGCACGATTCTCACCTCTCTCGCTCTCGAAGAGTGGGAGGAAAAGCTCAAGACATCTCACTTACCTGAAATGGTGTTTGGCGAGAATGCATTAGTCCTTAAACACTTGAGCAGCAACACTAAGATTCATTTTAACGCATTCGATGCACTAGCGGGCTGGAAGCAGGAAGGGCTTCCACCTGTTGAAGTTCCTGCTGCAGCACAATGGAAATTCAGGAGCAAGCCGTCCCAGCAGGTGATACTAGATTATGATTACACTTTTACGACGCCATACTGTGGAAGTGAAGTCGTTGAGAAAGACCAAGATGCGGTTGACGCAAAAGCTAATCCTGAGGGGGAAGCTAGTCTTCAGTGGGAGAACTGTGAAGAGCAGATTGATTTGGCTGCTCTCTCACTTAAAGAACCTATTCTCTTCTATGATGAGGTAGTTTTGTATGAAGATGAACTGGCCGACAATGGAGTCTCGCTTCTGACTGTGAAAGTGAGAGTCATGCCAAGTTCATGGTTCCTCCTCTTACGATTTTGGCTTAGAGTTGATGGTGTGCTTATGAGACTGAGAGAGACGAGAATGCATTATGTGTTTGGCAAAGGTGAGGCACCCACTGTTCTTCGTGAAAGCTGTTGGAGAGAAGCGACATTTCAGTCTCTATCTGCGAAAGGGTATCCTGTTGATTTAGCAGTCTATAGTGACCCTAGCTCCATCAGTCAGAGGCTTCCCGTGATTAAGCAGATAACACAGAAACTGAAGATCCCTCGTAAAGTG
